# Supplementary material for: Evidence for the acquisition of a proteorhodopsin-like rhodopsin by a chrysophyte-infecting giant virus
Source: J Virol. 2026 Aug 10;100(9):e00520-26. doi: 10.1128/jvi.00520-26 (PMC13595923; doi:10.1128/jvi.00520-26)
Supplement: Supplementary file 1 [file jvi.00520-26-s0001.pdf]

Supplemental Material for:

Evidence for the acquisition of a proteorhodopsin-like rhodopsin by a chrysophyte-infecting giant virus

Petra Byl[#, 1, 2, 3, *], Christopher R. Schvarcz[1, 2], Julie Thomy[1, 2], Qian Li[1, 2], Cori B. Williams[1], Kurt LaButti[3], Frederik Schulz[3], Kyle F. Edwards[2], Grieg F. Steward[#, 1, 2]

[1]Daniel K. Inouye Center for Microbial Oceanography: Research and Education, School of Ocean and Earth Science and Technology (SOEST), University of Hawaiʻi at Mānoa, Honolulu, HI, USA.

[2]Department of Oceanography, School of Ocean and Earth Science and Technology (SOEST), University of Hawaiʻi at Mānoa, Honolulu, HI, USA.

[3]DOE Joint Genome Institute, Lawrence Berkeley National Laboratory, Berkeley, CA. USA.

#Address correspondence to Petra Byl pbyl@umces.edu & Grieg Steward grieg@hawaii.edu

*Present address: University of Maryland Center for Environmental Science, Horn Point Laboratory, Cambridge, MD, USA.

List of contents:

1. Table S1
2. Figure S1
3. Figure S2
4. Figure S3
5. Figure S4
6. Supplemental References

Table S1. Noteworthy ChrysoHV genes grouped by function, showing their occurrence (tally), position (locus tag), and NCBI GenBank accession ID. The table includes various auxiliary metabolic genes, methyltransferases, glycotransferases, tRNAs, and ribosomal proteins.

Potential Function	Tally	Locus Tag	Genbank Accession ID
**Cell Wall Structure**			
N-acetylmuramoyl-L-alanine amidase	1	1143	XVM29061.1
**Electron Transport Chain**			
Cytochrome b5	2	242, 642	XVM28163.1, XVM28561.1
Flavinator of succinate dehydrogenase	1	538	XVM28459.1
Succinate dehydrogenase subunit	3	766, 767, 769	XVM28684.1, XVM28685.1, XVM28687.1
Prohibitin-like protein	1	927	XVM28845.1
**Glycosylation**			
Glycosyltransferase	15	127, 706, 726, 969, 970, 972, 973, 974, 976, 977, 979, 980, 982, 992, 1051	XVM28048.1, XVM28625.1, XVM28645.1, XVM28887.1, XVM28888.1, XVM28890.1, XVM28891.1, XVM28892.1, XVM28894.1, XVM28895.1, XVM28897.1, XVM28898.1, XVM28900.1, XVM28910.1, XVM28969.1
**Iron/Redox Related**			
2OG-Fe(II) oxygenase	1	750	XVM28668.1
Fe-S biosynthesis	1	775	XVM28693.1
Fe-containing alcohol dehydrogenase	1	968	XVM28886.1

**Light/UV Stress**			
Photolyase	3	95, 104, 530	XVM28016.1, XVM28025.1, XVM28451.1
Chlorophyll A-B binding protein	1	850	XVM28768.1
**Methylation**			
Methyltransferase	7	44, 113, 160, 684, 741, 988, 1131	XVM27969.1, XVM28034.1, XVM28081.1, XVM28603.1, XVM28660.1, XVM28906.1, XVM29049.1
**Phosphate Limitation**			
Phosphate starvation protein (phoH)	1	1007	XVM28925.1
**Sulfonation**			
Sulfotransferase	2	1035, 1046	XVM28953.1, XVM28964.1
**Tail Structure**			
Tail fiber domain-containing proteins	2	194, 986	XVM28115.1, XVM28911.1
**Transport**			
ABC transporter	7	94, 247, 713, 800, 920, 951, 961	XVM28015.1, XVM28168.1, XVM28632.1, XVM28718.1, XVM28838.1, XVM28869.1, XVM28879.1
Ammonium transporter	2	689, 1074	XVM28608.1, XVM28992.1
Porins	1	531, 650, 651	XVM28452.1, XVM28569.1, XVM28570.1
SemiSWEET sugar transporter	1	203	XVM28124.1

Urea transporter	2	1087, 1088	XVM29005.1, XVM29006.1
**Translation**			
Ribosomal protein S27a	1	1099	XVM29017.1
tRNAs	7	75, 76, 77, 78, 615, 616, 748	*NA*
Ubiquitin-60S ribosomal protein L40	1	536	XVM28457.1
**Rhodopsin**			
Heliorhodopsin	2	106, 938	XVM28027.1, XVM28856.1
Proteorhodopsin	1	101	XVM28022.1

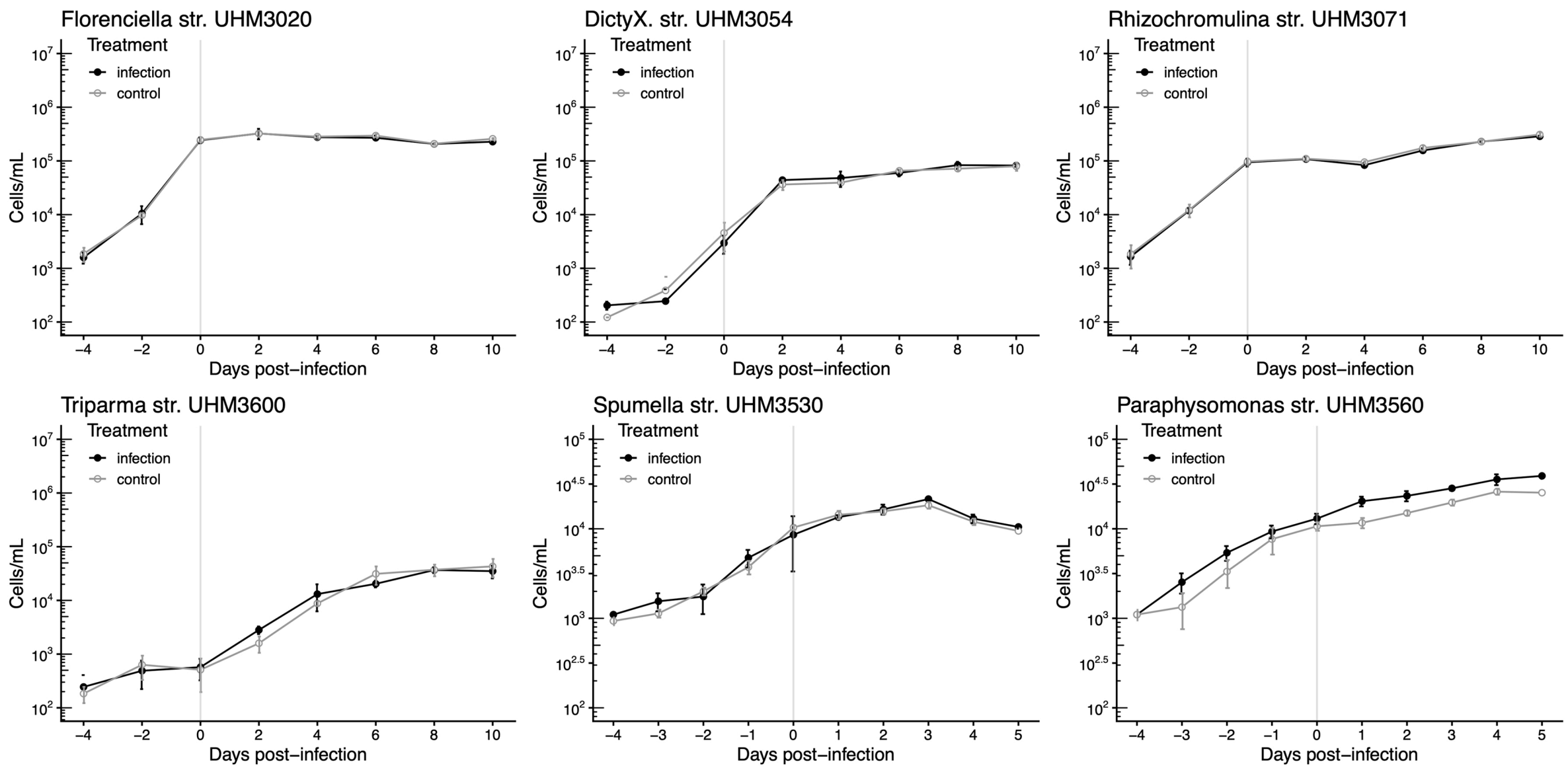

Fig. S1. Mixotrophic and heterotrophic protists resistant to ChrysoHV. Time series of protists isolated from Station ALOHA: *Florenciella* str. UHM3020, Dictyochophyceae Clade X str. UHM3054, *Rhizochromulina* str. UHM3071, *Triparma* str. UHM3600, *Spumella* str. UHM3530, and *Paraphysomonas* str. UHM3560 were challenged with raw ChrysoHV lysate at the point indicated by the vertical dashed line. Data for control cultures are shown in gray with open points and data for cultures inoculated with virus are shown in black lines with black-filled points.

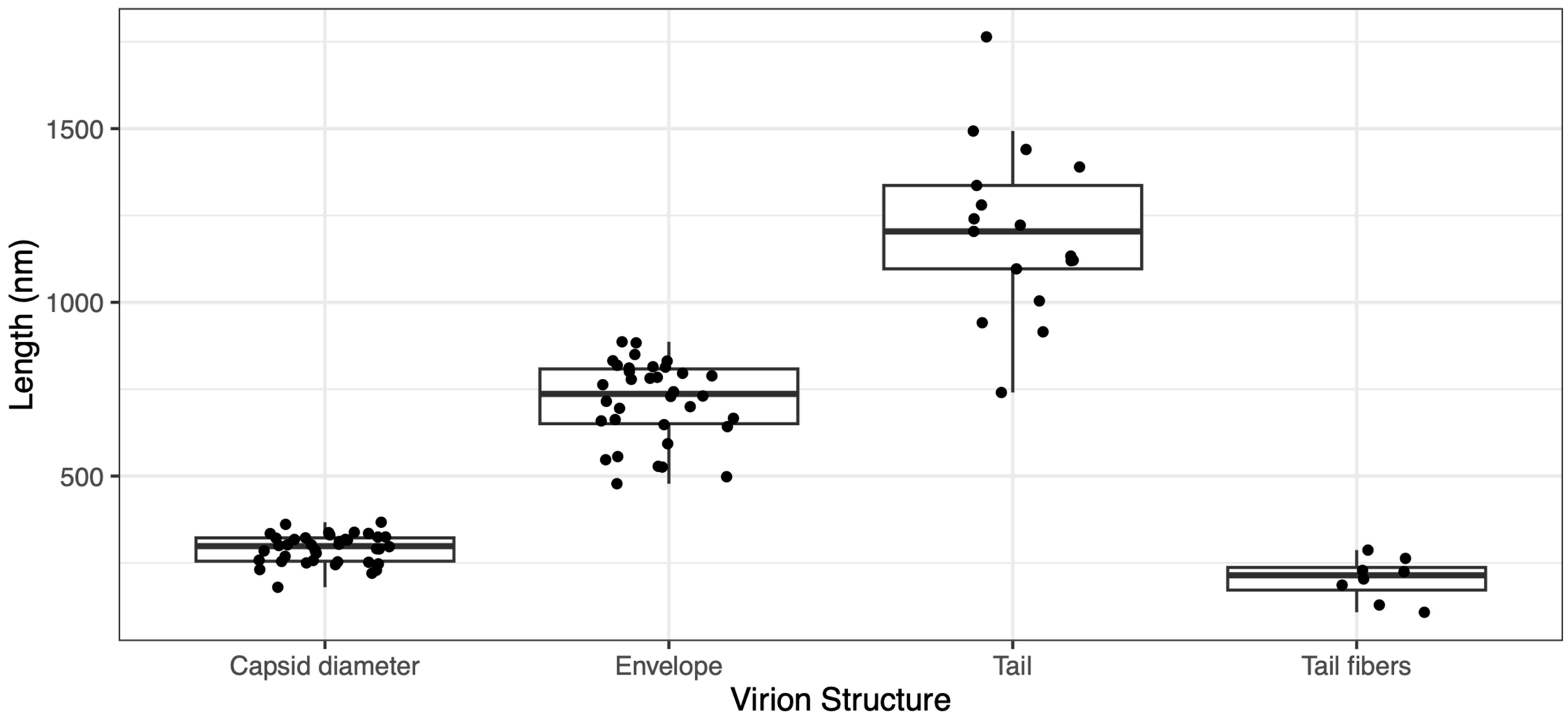

Fig. S2. Measurements of ChrysoHV virion features. Each dot represents a data point and each box denotes boundaries of the upper and lower quartiles of each measurement, and the thick, black horizontal line denotes the median value. The whiskers extend out to non-outlier minimum and maximum values.

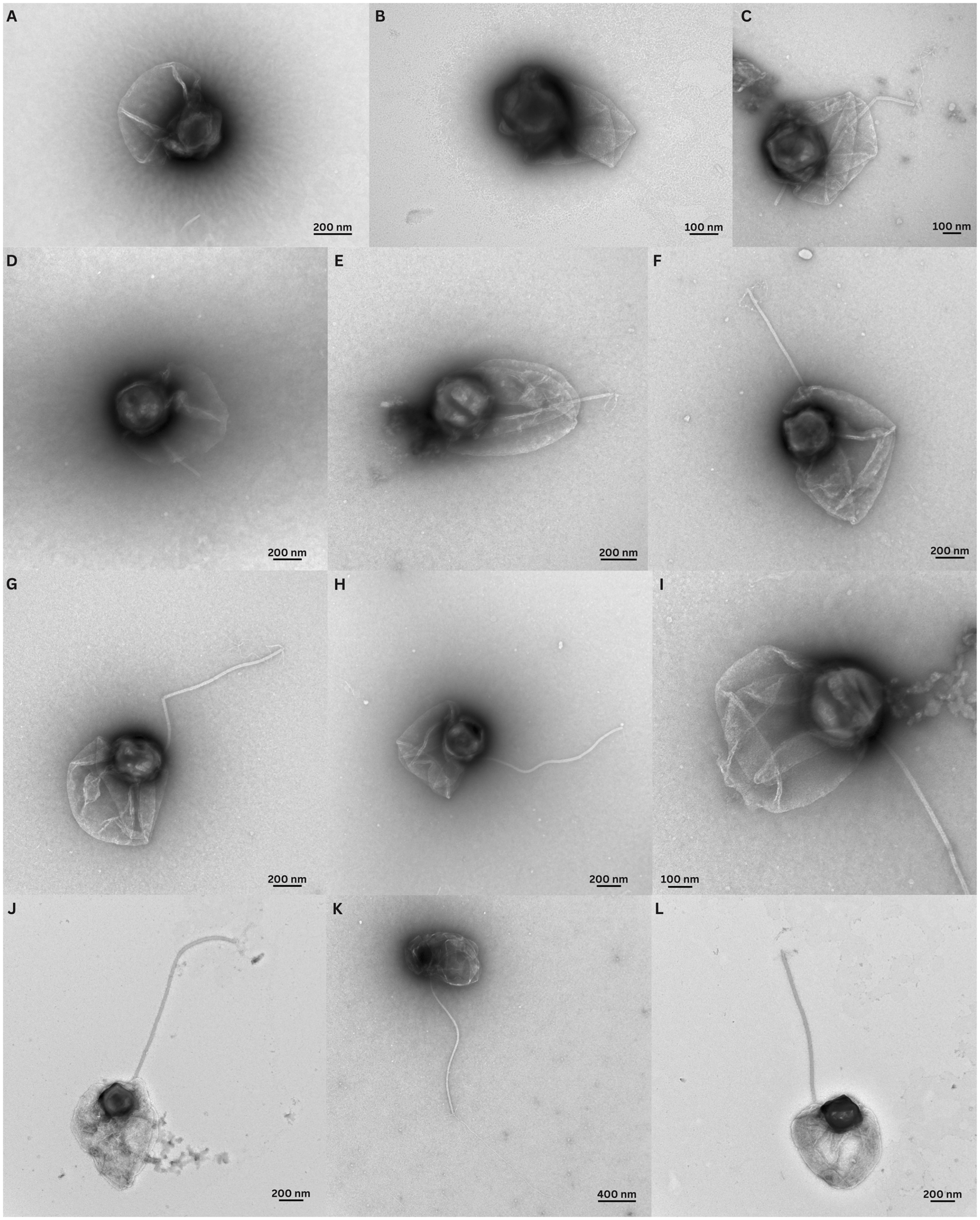

Fig. S3. TEM micrographs of ChrysoHV. The images show (A, B) non-tailed morphotypes propagated on chrysophyte Clade H UHM3501; (C, D, E) virions with the tail folded underneath the capsid and envelope (lysate from chrysophyte Clade H UHM3500); and (F-L) tailed morphotypes propagated on UHM3500.

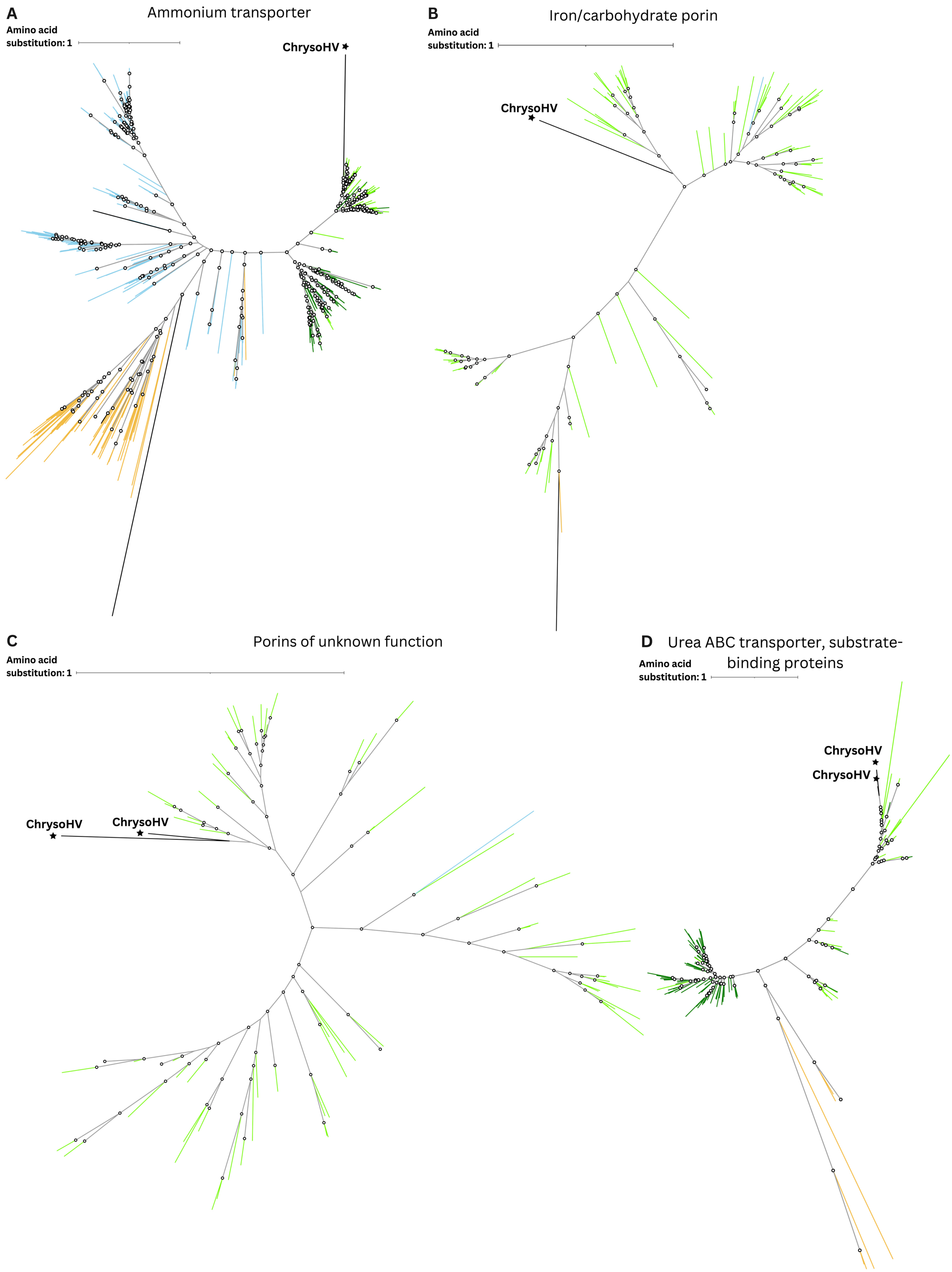

Fig. S4. (continued)

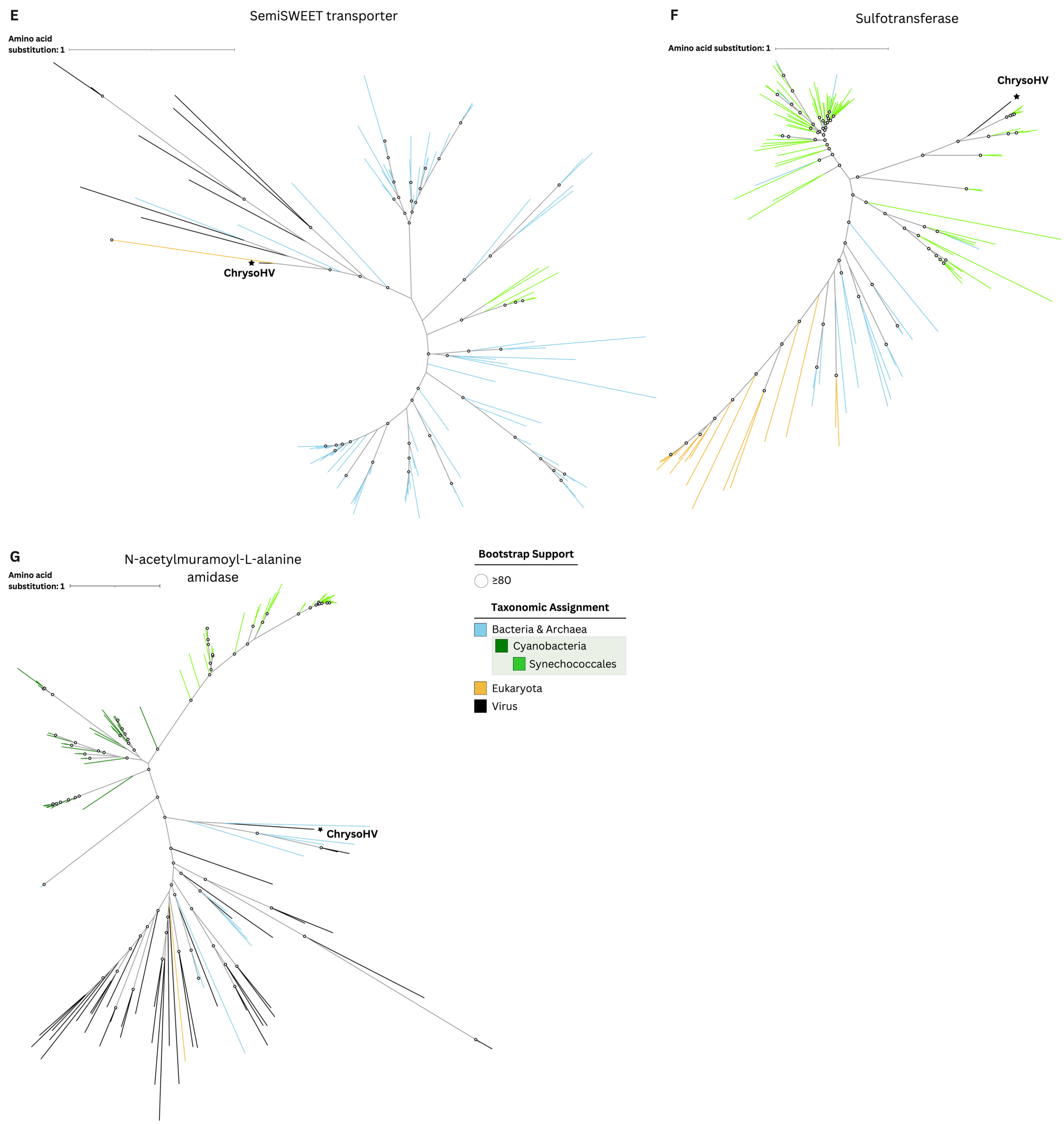

Fig. S4. Maximum likelihood phylogenies of cyanobacterial homologs found in ChrysoHV. The gene trees include an (A) ammonium transporter (XVM28992.1), (B) iron/carbohydrate porin (XVM28452.1), (C) porins of unknown function (XVM28569.1, XVM28570.1), (D) urea ABC transporter, substrate-binding proteins (XVM29005.1, XVM29006.1), (E) semiSWEET sugar transporter (XVM28124.1), (F) sulfotransferase (XVM28964.1), and (G) N-acetylmuramoyl-L-alanine amidase (XVM29061.1). The query sequences represent top BLASTp

(1) hits retrieved from the NCBI ClusteredNR database, NCBI Virus database, and Marine Microbial Eukaryote Transcriptome Sequencing Project (MMETSP) (2, 3), then quality filtered for a query coverage ≥ 50% and an E-value ≤ 1e-05. Not all gene trees contained viral or eukaryotic sequences that met these criteria. For closely related genes, such as the porins (C) and urea substrate-binding proteins (D), these top hits were dereplicated and combined into one tree. Hits to the same transcriptome were also dereplicated. The query sequences were aligned with MAFFT (L-INS-i; v7.520) (4), trimmed with trimAl (-gt 0.1; v1.4.1) (5); and the phylogeny was built with IQ-TREE best-fit substitution model and ultrafast bootstrap supports (-m TEST, -bb 1000; v2.2.6) (6), then annotated in iTOL (v6) (7). Branch color indicates sequence taxonomy with the order Synechococcales, which includes Prochlorococcus and Synechococcus, in bright green; all other cyanobacteria phylum representatives are shown in dark green. Other prokaryotic sequences are shown in light blue, and eukaryote-derived sequences are shown in yellow. Virus sequences are shown in black with ChrysoHV sequences labelled in bold font with a star symbol. Bootstrap support above 80% is indicated by a white circle. Branches for sequences YP_010670258.1 and Amphidinium_carterae_Transcript_38023|m.85619 were pruned from the porins of unknown function (C) and urea substrate-binding protein (D) gene trees because of their excessive length.

References

1. Camacho C, Coulouris G, Avagyan V, Ma N, Papadopoulos J, Bealer K, Madden TL. 2009. BLAST+: architecture and applications. *BMC Bioinformatics* 10:421.

2. Keeling PJ, Burki F, Wilcox HM, Allam B, Allen EE, Amaral-Zettler LA, Armbrust EV, Archibald JM, Bharti AK, Bell CJ, Beszteri B, Bidle KD, Cameron CT, Campbell L, Caron DA, Cattolico RA, Collier JL, Coyne K, Davy SK, Deschamps P, Dyhrman ST, Edvardsen B, Gates RD, Gobler CJ, Greenwood SJ, Guida SM, Jacobi JL, Jakobsen KS, James ER, Jenkins B, John U, Johnson MD, Juhl AR, Kamp A, Katz LA, Kiene R, Kudryavtsev A, Leander BS, Lin S, Lovejoy C, Lynn D, Marchetti A, McManus G, Nedelcu AM, Menden-Deuer S, Miceli C, Mock T, Montresor M, Moran MA, Murray S, Nadathur G, Nagai S, Ngam PB, Palenik B, Pawlowski J, Petroni G, Piganeau G, Posewitz MC, Rengefors K, Romano G, Rumpho ME, Rynearson T, Schilling KB, Schroeder DC, Simpson AGB, Slamovits CH, Smith DR, Smith GJ, Smith SR, Sosik HM, Stief P, Theriot E, Twary SN, Umale PE, Vaulot D, Wawrik B, Wheeler GL, Wilson WH, Xu Y, Zingone A, Worden AZ. 2014. The Marine Microbial Eukaryote Transcriptome Sequencing Project (MMETSP): Illuminating the Functional Diversity of Eukaryotic Life in the Oceans through Transcriptome Sequencing. *PLoS Biol* 12:e1001889.

3. Johnson, Lisa K., Alexander, Harriet, & Brown, C. Titus. (2018). MMETSP re-assemblies. https://doi.org/10.5281/zenodo.740440 (06 July 2026, date last accessed)

4. Katoh K, Standley DM. 2013. MAFFT multiple sequence alignment software version 7: improvements in performance and usability. *Molecular Biology and Evolution* 30:772–780.

5. Capella-Gutiérrez S, Silla-Martínez JM, Gabaldón T. 2009. trimAl: a tool for automated alignment trimming in large-scale phylogenetic analyses. *Bioinformatics* 25:1972–1973.

6. Minh BQ, Schmidt HA, Chernomor O, Schrempf D, Woodhams MD, Von Haeseler A, Lanfear R. 2020. IQ-TREE 2: New models and efficient methods for phylogenetic inference in the genomic era. *Molecular Biology and Evolution* 37:1530–1534.

7. Letunic I, Bork P. 2024. Interactive Tree of Life (iTOL) v6: recent updates to the phylogenetic tree display and annotation tool. *Nucleic Acids Research* 52:W78–W82.
